# Offerings and User Demands of eHealth Services in Spain: National Survey

**DOI:** 10.2196/42304

**Published:** 2023-05-18

**Authors:** Andrés Cernadas Ramos, Ángela Fernández Da Silva, Bran Barral Buceta, Ramón Bouzas-Lorenzo

**Affiliations:** 1 Department of Political Science and Sociology Faculty of Political and Social Sciences University of Santiago de Compostela Santiago de Compostela Spain; 2 Department of Sociology, Political Science and Administration, and Philosophy University of Vigo Pontevedra Spain

**Keywords:** Spanish eHealth, health policies, digital health, COVID-19, eHealth offers, eHealth demands

## Abstract

**Background:**

The accelerated development of information and communication technologies has made health care one of the pioneering fields in the incorporation of these tools. As new technologies have been applied, existing technologies have been sophisticated and improved and the concept of eHealth has expanded. However, these advances and expansion of eHealth do not seem to have served to adapt the supply of services to users’ demands; rather, supply seems to be governed by other variables.

**Objective:**

The main objective of this work was to review the existing differences between user demands and the supply of eHealth services in Spain and their causes. The aim is to provide information on the level of use of the services and the causes of the variation in demand for these services, which can be useful in correcting existing differences and adapting them to the needs of users.

**Methods:**

A survey, “Use and Attitudes Toward eHealth in Spain,” was applied by telephone to a sample of 1695 people aged 18 years and over, taking into account sociodemographic profile characteristics (sex, age, habitat, educational level). The confidence level was set at 95% and the margin of error was ±2.45 for the whole sample.

**Results:**

The survey results showed that the online doctor’s appointment service is the most frequently used eHealth service by users: 72.48% of respondents used this service at some point and 21.28% stated that they use it regularly. The other services showed significantly lower percentages of use, including “managing health cards” (28.04%), “consulting medical history” (20.37%), “managing test results” (20.22%), “communicating with health professionals” (17.80%), and “requesting a change of doctor” (13.76%). Despite this low usage, a large majority of respondents (80.00%) attach great importance to all the services offered. Overall, 16.52% of the users surveyed were willing to make new service requests to the regional websites, with 9.33% of them highlighting services such as “the availability of a complaints and claims mailbox,” “the possibility of consulting medical records,” and “the availability of more detailed information on medical centers (location, medical directory, waiting lists, etc).” Other outstanding requests (8.00%) were to simplify the procedures for using certain existing services.

**Conclusions:**

The data from the survey show that eHealth services are widely known and highly valued by users, but not all services are used with the same frequency or intensity. It appears that users find it difficult to suggest new services that might be useful to them in terms of demand for new services that do not currently exist. It would be useful to use qualitative studies to gain a deeper understanding of currently unmet needs and the possibilities of eHealth. The lack of access to and use of these services and the unmet needs particularly affect more vulnerable populations who have the greatest difficulty in meeting their needs through alternative means to eHealth.

## Introduction

### Background

In recent years, information and communication technologies (ICTs) have become a central issue on the political agenda [1]. The accelerated development of ICTs had a strong impact on the field of health care, making it one of the pioneering areas in the incorporation of these tools, leading to a renewal of health care systems [2,3].

The introduction of ICTs in the field of health care provided great impetus to the development of eHealth, a set of tools used in the health care environment ranging from prevention to treatment, including diagnosis and monitoring, as well as health management. eHealth led to cost savings in health care systems, and has improved the access, efficiency, effectiveness, and quality of clinical and management processes for all stakeholders [4-7].

As new technologies have been applied and existing technologies have become more sophisticated and improved, the concept of eHealth has expanded, incorporating new elements to its definition, such as telemedicine (ie, ICT-supported health care) through, for example, image digitalization or consultations via video calls [8] along with mobile health (mHealth) with the development of medical practices supported by mobile devices such as smartphones, personal digital assistants, tablets, or wireless patient monitoring devices [7,9]. However, these advances and expansion of eHealth do not seem to have served to adapt the supply of services to users’ demands; rather, supply seems to be governed by other variables.

Therefore, it seems that eHealth has not taken user demands into account, as it has not adapted the supply to these demands.

### Target Population of eHealth

Initially, the term *eHealth* was not promoted in academic settings; however, based on commercial logic, it can be assumed that the benefits generated by ICTs in other areas could also be generated in the health sector with similar results. This approach led to a change in the concept of the patient, passing from user to consumer [4].

eHealth was born from a goal of reducing costs and automating administrative processes, revealing an orientation that is more linked to productivity than to meeting the needs of users, while facilitating online procedures and services [1,10]. This trend is in line with the willingness of large corporations, which seem to be joined by public administrations [11,12].

Increases in investment have facilitated a considerable expansion of knowledge, as well as of technologies, techniques, skills, and resources. This made several achievements possible, including addressing health problems much more effectively [3], increasing quality and safety for both professionals and users [1], streamlining the performance of health systems, increasing the coresponsibility of individuals for their own health, and achieving greater efficiency and sustainability [13].

However, the purpose of using ICTs to support cost optimization has led to a decrease in investment in services [14] and the configuration of an eHealth model that is more closely linked to the interests and needs of health managers and professionals than to those of users [15].

### Current eHealth Development Level

The application of ICTs to the field of health may generate benefits for the actors involved (users, professionals, and managers), offering a space for information, a means of interaction, and a tool for the provision of services [16]. However, several factors can attenuate or slow down the development of ICTs in the health care field [17], including the distrust of some users of the preservation of their personal data [10,18-20], the lack of digital literacy of some population groups [21,22], problems of interoperability of clinical information systems [23], or the lack of common protocols for different health care systems [24].

In Spain, the implementation and development of eHealth continue to advance. The degree of development is not yet complete, showing asymmetries depending on the different services. For example, the telematic request for appointments and electronic prescriptions is fully implemented, whereas tools linked to digital images management or access to digital medical records have not yet been fully implemented [25].

Although the pandemic caused by the SARS-CoV-2 virus highlighted the potential of eHealth [26,27] and implied a significant change in the way users and health care professionals relate to each other [28-32], this shift has not brought about major changes or innovations, despite accelerating processes initiated previously.

Assessing users’ satisfaction with an eHealth service could lead to developing an approach based on satisfaction of expectations that may be modeled from the outset to reduce the gap between what is expected and what is obtained as much as possible, helping to avoid discouraging possible future use [33,34]. However, the different gaps initially formulated by Zeithaml et al [34] should be taken into account, as the work of Lankton and Wilson [35] showed that previous satisfaction strongly conditions the expectations of future use. Therefore, policy makers, and especially those responsible for the development and implementation of digital health, should ensure that they generate services that are fully satisfactory for users to encourage the use of online health services, since these results will generate positive expectations that will lead to greater use of online health services in the future. In some cases, the services offered are not sufficiently tested and run, with consequent frustration for users and professionals who do not see their expectations met. This may generate distrust in the system as a whole, making the use of eHealth services more difficult in the present and short term [36].

Accordingly, the main objective of this study was to analyze the existing differences between user demands and the supply of eHealth services in Spain according to the results of a population-based survey. The aim is to provide information on the level of use of the services and on the causes of the variation in their demand. The findings can also highlight other services that could be useful for users.

## Methods

### Study Design

To gather information on the eHealth services provided by public health portals in Spain, as well as the use made of these services by users and their unsatisfied demands, we applied the *Survey of Use and Attitudes Toward eHealth in Spain* (hereafter referred to as the eHealth Survey).

Results were obtained from a block of questions in the survey that allowed us to examine (in isolation) certain profiles of use and acceptance of eHealth according to social characteristics (age, sex, educational level, and habitat). This further enabled detecting mismatches between the supply and demand of services and suggesting the causes of such mismatches.

Prior to this, an extensive analysis of the recently published scientific literature on eHealth was carried out with the primary aim of identifying what services are currently offered in different countries in this post-COVID-19 era and to further investigate unmet user demands. In addition, to support the survey design, heuristic and user tests were carried out with the intention of defining the specific questions and categories for analysis.

### eHealth Survey Design

The survey was conducted through telephone calls and was supported by computer-assisted telephone interviewing technology. During the survey, opinions were sought on questions concerning access to the main services offered by the 17 regional health web portals: requesting medical appointments, access to the digital medical record, management of electronic prescriptions, digital imaging, and telemedicine. Other questions were also included to identify the services that are most valued by users and those that were missing.

The average length of the survey was 9.0 minutes, with a range of 5.7 to 12.3 minutes, depending on the existence of filter questions. Fieldwork was conducted between May 24 and June 21, 2018, throughout Spain, except for the African autonomous cities of Ceuta and Melilla.

Given the objectives of the research, the questions asked in the survey consisted of filters that determined two profiles: internet users and users of eHealth services. The first group comprises the second, whereas only the latter group could effectively assess the situation of eHealth services.

However, we were not only concerned with the opinion of those who use eHealth services. In fact, it is even more important, if possible, to identify the reasons why those who have the necessary means and knowledge to make use of these services do not do so.

Specifically, the first question made it possible to distinguish between those who browse and those who also access the main entry point for eHealth services in the regional web portal in Spain.

### Population

A sample of 1695 people legally residing in Spain who were aged 18 years or older was randomly surveyed. Telephone calls were made through the Infobel v16 directory during different time slots and days of the week. To guarantee adequate representativeness, the selection took into account sociodemographic profile characteristics—establishing quotas for sex, age, and habitat (the capital city and at least one city of each size were represented in all provinces)—with a confidence level of 95% and margin of error of ±2.45 for the sample as a whole (see Multimedia Appendix 1).

### Analysis and Interpretation

The information collected was stored in a coded database following the survey objectives and design. Preliminary results were corrected, standardized, and recoded for some variables to facilitate their statistical treatment. Weightings were applied to ensure representativeness at the state level according to Spanish national statistics institute data. Finally, a detailed analysis was carried out using the computer packages SPSS and STATA, from which the general opinions of the group of respondents were extracted, ranked, and their contribution to the research objectives was assessed.

Descriptive analysis was performed along with detection of critical profiles based on the sociodemographic variables, with the aim of examining the aspects that are associated with any detected differences between the services offered and the demands of the users.

### Ethical Considerations

The study was approved by a resolution of the Bioethics Committee of the University of Santiago de Compostela, dated January 12, 2018. The research technique (survey) was based on anonymization, and the data collected were processed using codes that did not identify the informant in any way. The management of contact data was carried out in accordance with the bioethical regulations of the University of Santiago de Compostela and in compliance with Spanish legislation on data protection (Spanish Law 15/1999 on the Protection of Personal Data), which was in force at the time the research was carried out. No financial compensation was provided to the respondents.

## Results

### Characteristics of Users of eHealth Portals

More than half of the population surveyed had never accessed their regional health portal (587/1103, 53.22%). When analyzing the data according to the variables used as descriptors (age, sex, education, and habitat), taking into account the age factor, the youngest group (18-24 years old) and those aged over 65 years reported connecting less to the health portals, with 61.07% and 67.69%, respectively, indicating that they do not access eHealth portals. By contrast, 32.18% of those in the internet users group indicated that they frequently connect to these portals. Among these, the age groups of 25-39 years and 40-65 years stand out, with a rate of 33.60% each.

There were no major differences detected by gender, although it should be noted that women use these services more frequently than men (34.05% vs 30.39%) and are generally slightly more frequent users of eHealth services than men (51.58% vs 54.77% reported having never accessed eHealth services).

There were differences noted regarding education level: those with university-level education reported more frequent access to these services (37.13%) than those with only secondary education (29.90%) or those with only primary education (28.41%). Among those with only secondary education, 56.36% stated they had never accessed these services, whereas 61.36% of those with primary education and 53.96% of those with university education stated that they had connected at least once.

Finally, with respect to habitat, there were differences between residents living in municipalities with more than 25,000 inhabitants and those with smaller populations (less than 5000 and between 5000 and 25,000 inhabitants): the former group accessed these services in a greater proportion (51.77% have never accessed compared to nearly 56.00% for the other two groups) and more frequently (33.48% reported using the services regularly compared to 30.00% for the other groups).

As anticipated, we determined it to be just as important to identify the motives of those who accessed eHealth services as determining the motives for those who have not accessed the services. Therefore, a specific block of questions was defined for the latter group. The main purpose was to identify the reasons for not having visited their respective health portal. To this end, a closed set of options was defined and the possibility of open answers was enabled (see Multimedia Appendix 2).

The main reason stated for not visiting health portals was that this need is covered by traditional means (45.98%). The youngest group (57.69%) gave this reason more often than respondents in older age groups (46.46%, 44.10%, and 40.00% for the age groups of 25-39, 40-65, and >65 years, respectively). The next most common reason given was disinterest, which was reported by 27.46% of those consulted, and reached up to 32.50% among older respondents.

Other indicated reasons for not visiting the portals included having alternative coverage, which reached 9.15% of the total and 15.00% among the older respondents, and the difficulties of using the network and this type of portal, as stated by 4.46% of all internet users and nonusers of health portals.

Other reasons given to a lesser extent were the perceived unreliability of the information provided by the websites, and the lack of privacy and security on the internet, accounting for less than 4% of the total responses.

Considering the gender variable, the main differences were that men reported more often than women that they meet their needs through traditional channels as a reason for not using the internet for health services by a difference of 7.33 points (49.55% vs 42.22%). Women displayed more diversity in their reasons for nonuse; 6.67% of women (compared to 2.23% of men) pointed to the difficulty of use as an obstacle, but there were also differences compared to men in the availability of other types of health care and in the consideration that information is not reliable.

Regarding educational level, several observations stand out. First, the association between having a university degree and the availability of alternative health care showed a significant difference with respect to the other educational levels (13.19% compared to 2.17% in those with primary education and 7.44% in those with secondary education).

Among those with primary education, significant differences were also observed with respect to the average. Among this group, 15.22% highlighted the difficulty of use and 8.7% indicated lack of confidence in the information. Likewise, the nonresponse rate to this question was 6.52%. In addition, up to 30.43% stated that they had no interest in this type of service format. Notably, half (50.00%) of those with secondary education stated that their needs are covered by traditional means, while 4.55% stating that they have difficulties in using this type of platform.

Finally, reviewing the data according to habitat, few differences were detected, highlighting a slightly greater lack of interest in these portals in larger cities (29.30% compared to approximately 25% in municipalities of up to 25,000 inhabitants).

### Reasons for Accessing eHealth Portals

The remaining survey questions were only asked of those who accessed the health portals. First, it was necessary to determine the reasons for accessing the services, followed by determining the most and least frequented services. These data (see Multimedia Appendix 3) show that visits to the regional portals are mainly aimed to perform or access some eHealth service or procedure, considering account accesses in general (63.16%) or the most recent visits (68.67%).

The next most common reason for visiting the portals was to search for information, with 29.28% reporting this reason in general and slightly less with respect to the last access (26.96%).

The third reason was to try to contact the health service, which was given for 5.43% of the respondents with respect to general access and 2.91% considering only the last access. The least common reason was to consult the employment section (0.82% in general and 0.73% for the last access), and other minor reasons.

In terms of age, there was a clear difference between the younger age groups (18-24 and 25-39 years) and the older groups (40-65 and >65 years), with the former showing a wider distribution of actions on the web portals compared to the latter.

Specifically, it is worth highlighting that 10.14% of respondents in the 18-24 years age group indicated that they usually access these portals to communicate with the health service. However, for the last access, this figure dropped to 1.64%, and only 8.33% of those in the older age groups indicated accessing the portal for this purpose the last time they had visited, which is a surprising finding.

There were no remarkable differences regarding gender or education, although it was noted that those with only primary education never accessed the portal to consult the employment exchange, which is likely linked to the level of training that is generally required for obtaining most jobs in this sector.

Finally, there were some notable findings with respect to differences in habitat. The majority of those accessing the portal for a job search were residents of large cities, whereas there was no access for this reason stated by people from intermediate or small habitats at any time (in general or the most recent occasion).

Another element to highlight is the significant percentage of access to communicate with the health service in small municipalities (up to 5000 inhabitants): 13.51% of the sample reported doing so in general and 7.58% for their last access. These proportions are almost three times higher than those for the next category of 5001 to 25,000 inhabitants (4.72% for general access and 2.48% for last access).

### Most Frequently Used Services on eHealth Portals

We next examined the most used services and their frequency of utilization (Multimedia Appendix 4).

The first element to note is that even among those who do use eHealth services, almost all showed an extremely high level of nonuse. The exception to this rule is for the booking or rescheduling of medical appointments. With the exception of this service, on average, almost 80% (79.96%) of the remaining services had never been used by users, whereas medical appointments had been used at least once by 72.48% of these respondents, followed by services of “health card management” (28.04%), “medical history consultation” (20.37%), “exam results management” (20.22%), “contact with health professional” (17.80%), and finally “request change of physician” (13.76%).

The only service that is used frequently is the request or change of medical appointment, which is used by 21.28% of people on a very regular basis. However, this common use of eHealth services is not as widespread. The remaining services hardly show frequent users and their pattern of use is much more sporadic. We further observed that usual or daily use is practically nonexistent, barely exceeding 2% of any of the patterns of access to the services.

With respect to age, patterns of access can be seen in which the percentage of use decreased as the age of the interviewee increases. This was the case with the use of health card management, requesting a change of physician, and contact with a health professional. For test results management and consulting medical background, the pattern was the same for the first three age groups, whereas those aged over 65 years showed higher percentages of use.

Finally, with regard to the main eHealth service, booking or rescheduling a medical appointment was particularly requested by people between 40 and 65 years of age (75.19%), followed by those between 25 and 39 (72.96%), those between 18 and 24 (67.24%), and finally those over 65 (52.00%) years old.

There were minor differences detected with respect to gender, with a marked imbalance in the service related to the management of the health card, which women used by 8.27 points less than men (23.75% vs 32.03%). For the other services, the proportions of use were quite even, although the pattern was slightly higher for men than for women.

With respect to educational level, when considering the ratings of people accessing eHealth services, the picture changes with respect to that described in Multimedia Appendix 3. Three characteristics could be observed: (1) the levels of use are more equal; (2) people with primary education continued to be the least likely to use the services (76.39% never use these services); and (3) on average, 71.46% of users with university education and 69.98% of those with secondary education do not access these services.

Finally, regarding the weight of the habitat factor, it could be observed that in municipalities with less than 5000 inhabitants, 68.94% or respondents have never used the services. This percentage increased to 70.26% in municipalities with larger populations and reached its maximum value (75.50%) in towns with between 5001 and 25,000 inhabitants.

In turn, we found that the smaller municipalities (less than 5000 inhabitants) make greater use of services such as health card management (35.94%), medical history consultation (28.13%), or test results management (25.40%).

### Most Important Services for Users

Beyond use of the service, the respondents were again asked about these services, but on this occasion were asked to state the importance they attributed to them, regardless of whether or not they made use of the services. That is, with regard to the importance attached by users to the services offered by the health portals, whether or not they have used them, information was collected through the question “And how important is each of these services to you?” (see Multimedia Appendix 5).

Regardless of the number of services used, the respondents tended to consider all of the services offered to be very important: more than 80% of the sample, on average, gave all the services presented some or a great deal of importance (63.18% stated “lots of” and 17.55% stated “some” importance), while the percentage of people who gave the services a rating of “little” or “no” importance did not exceed 10% in each case.

When analyzing each service separately, requesting and managing appointments was the most highly valued service (90.32%), followed by consultation of exam results (84.24%), health card management (81.14%), contact with health professionals (79.73%), consultation of medical records (76.89%), and requesting a change of physician (72.05%).

By age, the importance attributed to the range of services decreased with increasing age. By gender, there were hardly any differences between women and men in terms of the highest level of importance attached to services, at 81.74% and 79.77% respectively.

As the level of education increased, so did the importance given to all services offered: thus, people with university education gave an average of 86.16% importance to all the services, compared to 76.49% of people with secondary education and 74.19% with primary studies.

Focusing on habitat, there was no clear pattern. On average, people residing in municipalities with between 5001 and 25,000 inhabitants attached the most importance to the services offered as a whole (82.34%), followed by people in municipalities with more than 25,000 inhabitants (80.54%) and municipalities with less than 5000 inhabitants (78.88%).

### Mismatch Between User Demands and Offerings of eHealth Portals

Finally, to try to address the main objective of this study (*what do users expect and what does the National Health System offer them*?), the population surveyed was asked about the other services they would like to find in their regional health portals. To this end, a first question was established that filtered out, among the users of eHealth services, those who considered that there were services they would like to receive. Among the users surveyed, 16.52% were willing to make new service demands to autonomous websites. When this question was asked in an open-ended manner, the results shown in Table 1 were obtained.

**Table 1:** Responses to the survey question: “What service or information are you missing?”

Services	Responses, n (%)	Valid responses, %
Complaints/claims mailbox	7 (8)	9
Possibility of consulting medical history	7 (8)	9
Possibility of consulting information on the centers (medical direction, specialists, waiting lists, services, etc)	7 (8)	9
Simplify the process of booking/changing an appointment	6 (7)	8
Preventive recommendations/information on specific diseases	6 (7)	8
Request appointment with experts/nurses	5 (6)	7
Most user-friendly website with all the services it offers	5 (6)	7
Being able to consult waiting lists	4 (5)	5
Possibility of accessing the results of medical exams	4 (5)	5
Expand evening services (eg, appointments, exams)	4 (5)	5
Possibility of checking physicians’ availability (eg, schedules, vacations, hospitals)	4 (5)	5
Possibility of having medical consultations by mail/24 h	3 (3)	4
Book vaccinations/notifications about vaccination schedules	3 (3)	4
Suggestion box	2 (2)	3
Renew electronic prescriptions	2 (2)	3
More information and possibility of performing procedures/consultations with experts	2 (2)	3
Possibility of consulting information on pharmacies	2 (2)	3
Reminders of consultations	1 (1)	1
Management of sick leave/medical reports	1 (1)	1
Don’t know	11 (13)	Not applicable
Total	86 (100)	1

These data show that three requests shared the highest frequency (9%): availability of a mailbox for complaints and claims, the possibility of consulting medical records, and the availability of more detailed information on the centers (eg, medical directory, centers, waiting lists).

Other outstanding requests (8%) were to simplify the procedures for using certain services such as changing doctors or consulting medical records; to have more specific and extensive information on certain diseases; to have information on centers, professionals and their schedules, vaccination schedules, and pharmacies; and to be able to schedule appointments with experts or consult waiting lists.

Some of these demands may be covered by the offers made by some regional health services (eg, requesting an appointment with a nurse) or may even be included in the portfolio of services although users are not aware of them or do not have the appropriate permissions to access them (consultation of medical records, access to test results, complaints and suggestions mailboxes).

In turn, as can be seen in Table 1, the population consulted indicated more than 20 other services or information content that was lacking less frequently than those mentioned above, including preventive recommendations, having medical consultations by email, renewal of electronic prescriptions, management of sick leave reports, and a suggestion box or appointment reminders.

It should be borne in mind that the demands expressed may be conditioned by prior expectations, which in turn may tend to be closely related to certain services provided in other regions and for which the respondents have references.

Likewise, many of the demands were not related to new services or information but were rather more related to the simplification of procedures, improvements in access, ease of use, and search for services.

The small number of respondents who proposed demands overall, despite identifying shortcomings in the current offerings, could be due to the fact that many of the aspects surrounding eHealth services have technical connotations (ie, interoperability of systems, collection and transmission of images, teleconsultation and telecare, remote monitoring using wearables) that would make it difficult for people with a low or medium level of education to make their demands explicit.

## Discussion

### Principal Findings

New applications of telemedicine are being discovered all the time. Devices such as cell phones are replacing personal computers as connectivity support, so that mHealth has been enjoying significant development in recent years, mainly in preventive health [37,38]. It is believed that such tools may also have great potential for monitoring patients and chronic diseases [39,40], as well as in health promotion and in the collection of useful data for the system and for health care itself [41].

Other studies also reported the promising results that online programs are achieving in mental health [42], especially among professionals and younger users [43] or focused on self-isolating patients [44].

In preventive actions, screening, diagnosis, treatment, or follow-up, eHealth resources have come to be used extensively and on a daily basis [45], which has contributed to an improvement in the follow-up and information offered to users.

These advances are already known to some users; as they become more widely known and disseminated, their demand should also increase. For this reason, the conception of eHealth services is an important challenge for policy designers and implementers who should consider the needs and demands of the system’s users in their decision-making processes [46].

In addition to the adequacy of supply to demand and the satisfaction of expectations, another relevant condition to encourage greater use of online health services is the improvement of security. This is an aspect repeatedly considered to be the element of greatest concern among patients who have used online health services, especially when taking into account the management of data collected within the digital health record (DHR). Users argue that access to DHRs should be more protected [47] and should be limited exclusively to medical staff and not extended to other health care professionals such as nurses, pharmacists, or laboratory personnel. However, considering the results, it is also important to ensure that users are aware of and have access to their DHR, given that low levels of use may be an indication of a lack of access.

### Matching Supply and Demand for eHealth Services

The data collected from the survey show that, despite more than two decades of eHealth implementation, a sufficient match between supply and demand has not been achieved, as the results obtained show that users demand more than 20 services that they consider useful but are not currently being provided on a widespread basis. If current dynamics and trends continue, it would be difficult to achieve such a match, which seems to require a specific approach by eHealth policy makers and implementers.

Regardless of the specific technology considered, its introduction into a health care system should be preceded by an evaluation with well-defined criteria to demonstrate its efficacy, safety, and quality, and even supported by cost-effectiveness or efficiency criteria. These criteria imply the need to generate evidence showing feasibility, usability, acceptability, and cost until other formal and methodologically consistent studies that can provide evidence on cost-effectiveness are conducted [9].

More investment is needed in digital infrastructure, greater interoperability of the system, incentives for health professionals to promote its use, and a focus on people and outcomes. Simultaneously, services should be designed around users and their needs, medical teams need to be equipped with new skills—especially around digital health—to better respond to patients’ needs [48], and the incorporation of stakeholders in the design of online services should be increased [49]. Despite the fact that a large part of telemedicine is going to target people with disabilities, telemedicine is not designed with only those people in mind [50].

Moreover, some of the errors or shortcomings observed with respect to access and usability of services can be partially corrected by continued technological advances [51,52].

These advances should be accompanied by progress in terms of effectiveness, efficiency, accessibility, quality, and equality of treatment, along with greater capacity to measure results [53]. This requires consensus for implementation as well as leadership, since with a lack of budget and qualified personnel, the excessive reluctance of professionals to transition between the old and new care system [54] may generate rejection and resistance on the part of users. Thus, if responsible technologies are to be designed at the service of the citizenry as a whole, it will be necessary to integrate into the debate the various actors involved in digital health, and to establish mechanisms for monitoring and public scrutiny that provide transparency and legitimacy to these processes [55].

### Supply and Demand for eHealth and the Digital Divide

The social and digital divide remains in force and has more and more edges. The gap between the excluded and the included is widening [56] and this fragmentation of the system leads to unequal access and coverage [57], resulting in a possible replacement of medical staff by managers, administrative, and computer personnel [58], which gradually dehumanizes the user and the medical practice. Even the professionals recognize the importance of establishing deontological criteria for eHealth [59].

As some authors have pointed out [60], the digitalization of health care based on exclusive capitalist criteria puts at stake the survival of a health care model that enjoys a broad consensus, which has served to eradicate many diseases and has significantly increased life expectancy. As highlighted by Cometta [61] and others [62-64], the introduction of new technologies in the health sector risks increasing inequalities and endangering democracy by promoting forms of monitoring and social control.

If market logics are not brought under control and eHealth is not humanized to make it more social and inclusive, the health professions, as a space for inclusion and solidarity as they are known today, risk disappearing.

### Digital Health in the Post-COVID-19 Era

It is clear that the pandemic caused by COVID-19 has been a major boost for telemedicine in particular and for eHealth in general. Teleconsultations increased dramatically [65], which significantly contributed to reducing hospital pressure and the overload on health care systems by detecting, diagnosing, and monitoring COVID-19 from home [28,29] and also helping to reduce the risk of contagion [27].

In that period, national health services in different countries adopted telemedicine solutions as an alternative to face-to-face consultations. Some systems were not ready, as professionals were not trained/informed and had difficulties in accessing users’ medical records due to problems of interoperability of systems and applications, lack of training, the time required to use eHealth, or the prioritization of the search for immediate returns on investment [66].

It could be expected that some of the services demanded by users would be implemented during the COVID-19 pandemic, either because of the trend evolution of the digitalization of health care or because the pandemic could represent an important qualitative and quantitative leap in the use of technological tools to address the different challenges posed to health systems as a whole [67]. However, what is happening is rather an intensification of the use of the services that eHealth was already offering, which were included in the survey, rather than the implementation of new services in this way [68].

The pandemic has served to reduce certain resistance to the use of technology in attention and care. However, as observed in the results of this study and in previous research, users and some professionals continue to prefer face-to-face consultations, especially patients who are less familiar with technology and professionals who fear that medicine will become dehumanized and that professional practice will be reduced to the mere collection of data for biomedical studies [61].

### Study Limitations and Proposals for Improvement

The present work is a rigorous and well-documented study with first-rate information and data, both because of the research technique used and the extensive review of the literature carried out; nevertheless, it is believed that there are some aspects that could be improved.

In addition to the survey, it would be advisable to complement the quantitative data obtained with other qualitative information that could be obtained through other techniques such as interviews or focus groups.

It would also be convenient to collect and analyze the eHealth services offered in other countries in greater detail by including them in a database that would then allow us to compare the different health services with respect to their content, use, and valuation by users or citizens.

### Future Lines of Research

Since we are dealing with user demands and expectations in relation to eHealth services on the one hand and the necessary and constant evolution of the supply of these services on the other hand, it would be necessary to periodically update this information to be able to adapt the supply of these services to the changing demands of the population.

Given that users may not be aware of the full potential that new technologies offer in the provision of online health services, it would be a good idea to reflect on these potentialities and pass them on to users so that they can evaluate the services that seem most useful for addressing their needs.

It would also be necessary to look more closely at the services offered and others that could be demanded if they were offered, and to determine the reasons that these desired services are not being offered.

### Conclusions

The survey data show that the supply of eHealth services is not adapted to or does not currently meet the demands of users. It is also clear from these data that the services offered are widely known and well appreciated by users, although not all of them are used with the same frequency or intensity. This insufficient match between supply and demand for services may be due to the low or nonexistent presence of users in the process of designing eHealth policies, which mainly affects more vulnerable populations (eg, those with chronic diseases, older adults with mobility difficulties, those living in rural areas far from health centers), as they will face the greatest difficulties in covering their needs by alternative means. It might be expected that the COVID-19 pandemic, during which many health services were only provided telematically, would be used by eHealth to offer a wider range of services to users; however, as can be seen from the recent literature, both in Spain and in neighboring countries, the range of services has not been extended during this period but rather the use of existing services has been intensified.

## Appendix

Multimedia Appendix 1 Sampling errors.

Multimedia Appendix 2 Reasons for nonuse of eHealth portals.

Multimedia Appendix 3 Reasons for accessing eHealth portals.

Multimedia Appendix 4 Frequency of access to eHealth services.

Multimedia Appendix 5 Importance ratings of various eHealth services.

## Abbreviations

DHR: digital health record
ICT: information and communication technology
mHealth: mobile health

## Data Availability

The survey data will be available in a public repository [69].
