# Supplementary figures and images for: Offerings and User Demands of eHealth Services in Spain: National Survey
Source: J Med Internet Res. 2023 May 18;25:e42304. doi: 10.2196/42304 (PMC10236285; doi:10.2196/42304)

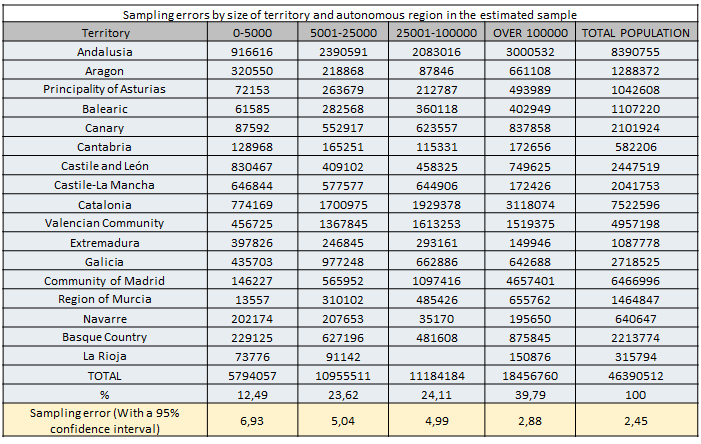

Supplement: Multimedia Appendix 1 [file jmir_v25i1e42304_app1.png]

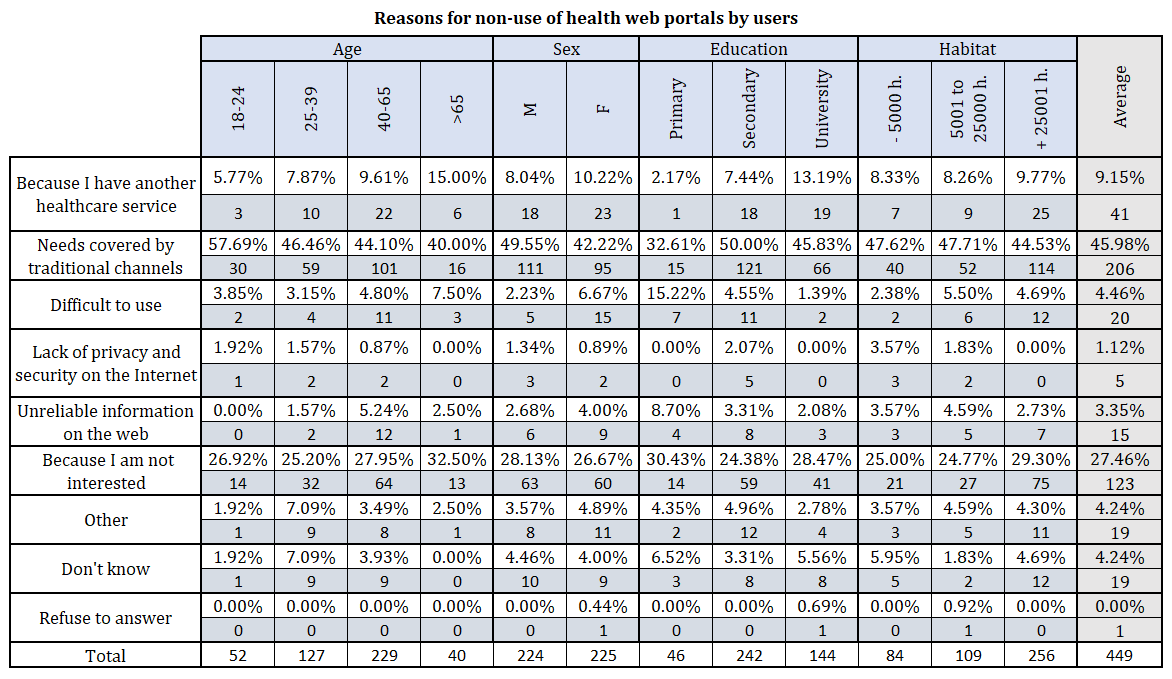

Supplement: Multimedia Appendix 2 [file jmir_v25i1e42304_app2.png]

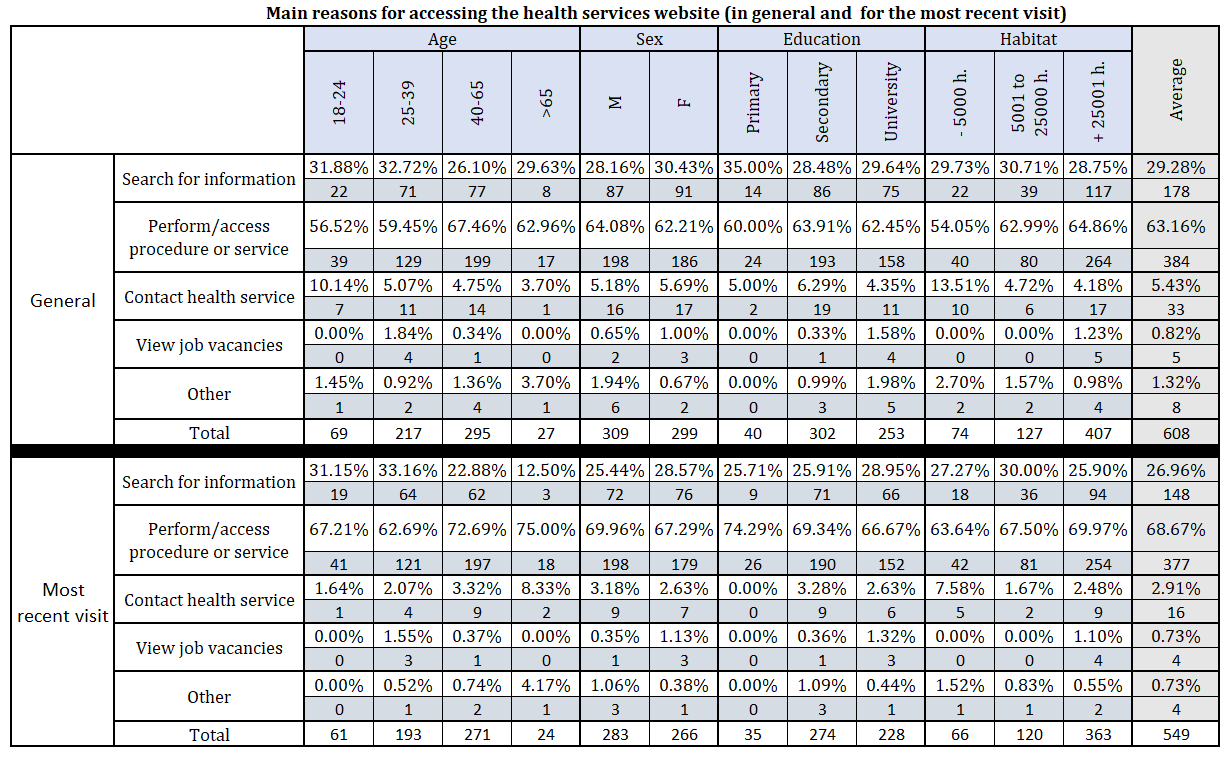

Supplement: Multimedia Appendix 3 [file jmir_v25i1e42304_app3.png]

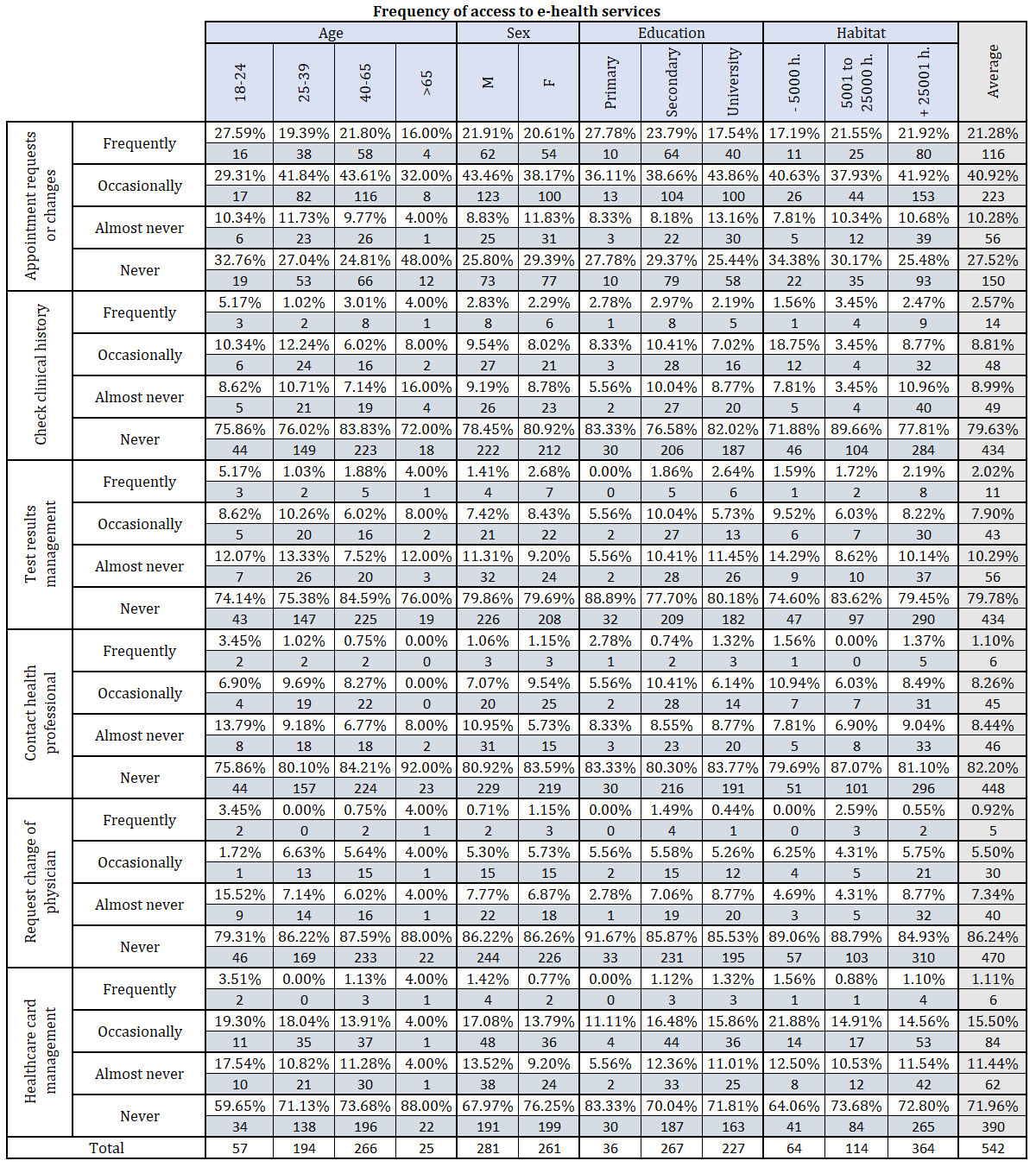

Supplement: Multimedia Appendix 4 [file jmir_v25i1e42304_app4.png]

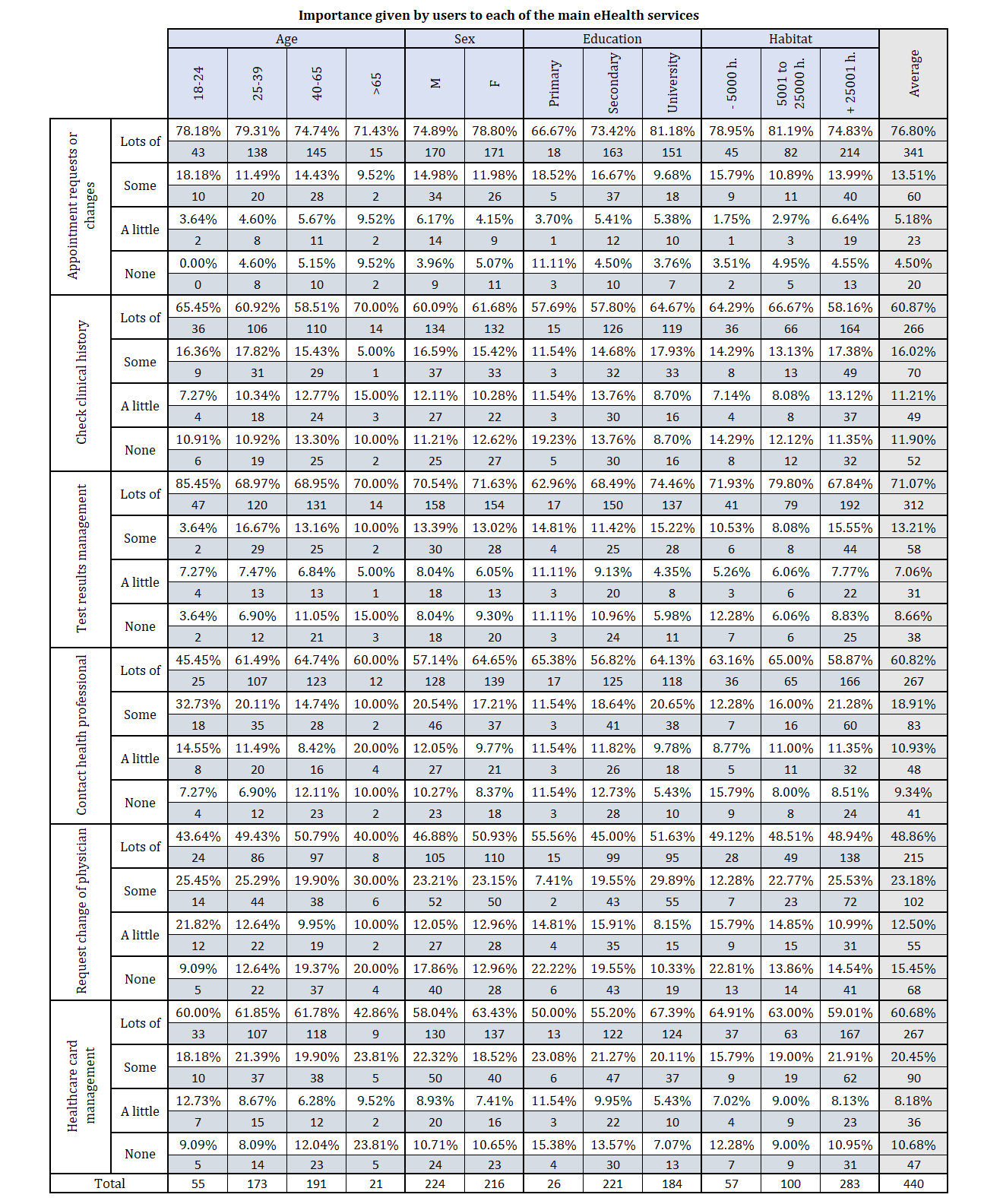

Supplement: Multimedia Appendix 5 [file jmir_v25i1e42304_app5.png]
